# Factors influencing public and private healthcare utilisation in Uganda

**DOI:** 10.4314/ahs.v23i3.83

**Published:** 2023-09

**Authors:** Medard Turyamureba, Bruno L Yawe, John Bosco Oryema

**Affiliations:** Makerere University, College of Business and Management Sciences

**Keywords:** Utilisation, multinomial logit, health care provider, policy, Uganda

## Abstract

**Background:**

In Uganda, health care utilisation remains very low despite a number of government reforms that have been implemented in the health sector since the 1990's such as decentralization and removal of user fees in public health facilities among others.

**Objective:**

To examine the factors influencing public and private health care utilisation in Uganda.

**Methods:**

The study used cross sectional data from the Uganda National Household Survey collected between July 2016 and June 2017. Anderson's conceptual framework was used to identify explanatory variables associated with choice of health care providers and a multinomial logistic regression model was estimated.

**Results:**

Out of the 17,912 individuals who sought care, 36% used a government facility, 60% used private facility while 4% had self- care/treatment. The results show that out of pocket health expenditure, age, level of education, marital status, residence, and type of illness significantly influenced choice of public healthcare providers. Similarly, utilisation of private healthcare providers was associated with household welfare, level of education, residence, marital status, illness days, and type of illness.

**Conclusion:**

The findings highlight the need for a national health insurance scheme to reduce out of pocket payments for health care and enable the poor and vulnerable patients visit the modern health facilities.

## Introduction

Many countries including Uganda continue to focus on promoting health care utilisation. In the last three decades, Government of Uganda has implemented a number of reforms in the health sector aimed at improving financing and delivery of quality healthcare services. These included: decentralization of health service delivery, autonomy of public hospitals as well as introduction of health financing reforms like community-based health insurance and removal of user fees in public health facilities (Republic of Uganda, 2015b; Republic of Uganda, 2010). In 2001, the government removed user fees in all government health facilities except at hospital level where a dual system exists 1.

However, health outcomes are still not impressive. Maternal mortality and child mortality rates remain very high at 336 per 1,000 live births and 64 per 1,000 live births respectively far above the sustainable development goal targets 2. Additionally, many Ugandans still experience very high out of pocket expenditure on health and contributes 41% of the total health expenditure 3. This not only limits access to health care but also creates financial risks especially for the poor who allocate much of their scarce resources to healthcare.

In Uganda, healthcare services are provided by both public and private health care providers. In 2018, Uganda had a total of 6,937 health facilities of which 45% were government owned, 15% were private and not for profit, and 40% were private for profit 4. In 2016/17, 83% of the individuals who fell sick or were injured sought health care and 13% did not seek health care 5.

Although studies indicate that patients are price sensitive, many Ugandans prefer private health facilities to less expensive government health facilities. In 2016/17, 33% of the individuals who fell sick or were injured sought care from public facilities, 48% thought care from private facilities while 14% sought care from pharmacies 5.

This is inconsistent with the objectives of the policy on abolition of user fees. This has implications for policy reforms on reducing the cost of health care or providing sustainable health care financing alternative. Therefore, this study sought to examine factors that influence public and private healthcare utilisation in Uganda.

## Methods

### Data source

The study used data from the 2016/2017 Uganda National Household Survey (UNHS) conducted by Uganda Bureau of Statistics. The survey took place between July 2016 and June 2017. It is the most recent available data set with indicators of healthcare provider and a range of demographic, social and economic variables. The survey used a two-stage stratified sampling. At the first stage, enumeration areas were grouped by district and rural-urban location which were then selected using probability proportional to size. At the second stage, households were selected using systematic sampling. The sample was a national representative, since it was drawn from all the districts.

A total of 1750 enumeration areas were selected and targeted to interview 10 households per enumeration area. Out of a sample of 17,500 households, 15,672 households were interviewed giving a response rate of 91%. The unit of analysis in this study was individuals who fell sick or were injured 30 days preceding the survey.

### Dependent variable

The dependent variable was healthcare provider used by patients. Different healthcare providers were grouped in to three categories namely: (1) self-care / self-treatment; (2) public / government; and (3) private. Self-treatment included use of drugs available at home and home-made medicines including roots, herbs, and drugs purchased from shops and from the market.

### Independent variables

Anderson's behavioural model of health care utilisation was adopted to guide selection of variables that may influence choice of a healthcare provider. According to this model, these factors are classified into predisposing factors (socio-cultural characteristics), enabling factors (logistical aspects of obtaining care), and need factors (immediate cause of healthcare service use or severity of illness or incapacity) 6.

Predisposing factors included in this study were age of the patient in completed years, gender, marital status, and education level. Sex was a binary variable with male and female categories while marital status was classified as either married or not married. Education level was classified into four groups; no formal education, primary, secondary and post-secondary education.

Enabling factors included in the analysis were: cost of care, household welfare as a proxy income, distance to the health facility, ownership of health insurance, residence (rural/urban), type of employment, household size, and region. Out-of-pocket health expenditure was used as a proxy for cost of health care. Distance in kilometres (kms) to the healthcare provider was categorised into four groups; less than 3kms, 3 to less than 5kms, 5 to less than 8kms and 8kms and above. Health insurance status was a dummy with having no health insurance cover as base category. Likewise, type of employment was categorised into three groups: substance farmer or unemployed, salaried, and self-employed. Region variable was included to capture geographic effects of central, eastern, northern, and western regions. Also, age and gender of the household head were included.

The need factors included illness days measured by number of days an individual was restricted from normal activities and type of illness which was categorised into four: minor/ fever, severe/chronic, injury, and other illness.

### Statistical analysis

All analyses were performed using STATA version 14.0. Distribution of each explanatory variable presented by healthcare provider (self-treatment, public, and private). A multinomial logistic regression model was then estimated using “self-care /treatment” as the reference category to examine factors associated with utilisation of public and private healthcare. In the Model 1 used all possible explanatory variables while in model 2, variables related to household head (age and sex) were dropped but introduced age-squared to check for existence of a non-linear effect of age.

Many empirical studies have used multinomial logit to study healthcare utilisation 7–9 because it's easy to apply compared to the multinomial probit model. However, it requires that the assumption of ‘independence of irrelevant alternatives’ (IIA) is satisfied 10,11. The Hausman's specification test was used to test if IIA assumption holds. The test results showed evidence for the null hypothesis in model 2 but not model 1. Therefore, model 2 was preferred since there was no violation of the IIA assumption.

## Results

### Descriptive analysis

Out of the 17,912 patients who sought care, 36% used public healthcare providers, 60% used private healthcare providers while 3% had self-care or did not consult a modern healthcare provider. Table 1 shows that the average cost of health care was lowest (2,698,700/=) for government healthcare providers compared to other providers. This reflects reality of subsidised or free healthcare services in government facilities. The average age of patients for private healthcare providers was 30 years, compared to 23 and 21 years for self-treatment and government respectively. The proportion of patients who had health insurance in private facilities (1%) was twice that of patients in government facilities (0.5%). Regarding the level of education, majority of patients who sought care had primary education indicated by 63%, 67%, and 67% for self-treatment, government and private healthcare providers respectively.

**Table 1:** Summary statistics by the type of healthcare provider

Variable	Self-treatment		Government		Private		Min	Max

	N	mean	N	mean	N	mean		
Cost of care ('00)	271	35,904	4,265	26,987	10,475	30,909	0	60,000
ln-welfare	314	11.188	6,837	11.079	10,672	11.375	8.59	15.14
Age	309	22.88	6,619	20.79	10,283	20.27	0	105
Age-squared	309	948.37	6,619	806.11	10,283	733.29	0	13,225
Illness days	315	5.61	6,849	4.90	10,705	4.22	0	30
Health insurance	315	0.010	6,849	0.005	10,705	0.010	0	1
**Distance**								
3 to <5kms	315	0.089	6,849	0.228	10,705	0.117	0	1
5 to <8kms	315	0.044	6,849	0.102	10,705	0.045	0	1
8kms and above	315	0.121	6,849	0.103	10,705	0.067	0	1
**Education**								
Primary	221	0.634	4,778	0.670	8,171	0.601	0	1
Secondary	221	0.172	4,778	0.174	8,171	0.208	0	1
Post-secondary	221	0.032	4,778	0.026	8,171	0.053	0	1
Sex (1 if female)	315	0.543	6,849	0.533	10,705	0.526	0	1
Married	309	0.295	6,619	0.269	10,283	0.301	0	1
Urban residence	315	0.184	6,849	0.243	10,705	0.305	0	1
**Type of illness**								
Chronic	315	0.140	6,849	0.155	10,705	0.147	0	1
Injury	315	0.102	6,849	0.061	10,705	0.058	0	1
Other illness	315	0.378	6,849	0.202	10,705	0.212	0	1
**Type of employment**								
Salaried	314	0.182	6,837	0.187	10,672	0.219	0	1
Self-employed	314	0.487	6,837	0.353	10,672	0.418	0	1
**Region**								
Eastern	315	0.200	6,849	0.279	10,705	0.273	0	1
Northern	315	0.295	6,849	0.383	10,705	0.252	0	1
Western	315	0.152	6,849	0.188	10,705	0.198	0	1
Household size	314	5.2	6,807	5.6	10,744	5.6	1	23
Sex of h/hold head	314	0.637	6,807	0.676	10,744	0.733	0	1
Age of h/hold head	314	44.4	6,807	44.4	10,744	42.0	14	101

*N=Number, Min= Minimum, Max=Maximum, h/hold= household*

**Source:** Authors' computation

### Multivariate analysis

Model 2 of Table 2 shows that the cost of healthcare significantly influenced choice of public healthcare provider and not private health care providers. Relative to self-treatment, the odds ratios of using public healthcare providers reduced significantly with an increase in out-of-pocket health expenditure. A similar trend was seen for health insurance. On the other hand, household welfare as a measure of household income positively influenced use of private health facilities relative to self-treatment (OR=1.38; 95%CI=1.10-1.74).

**Table 2:** Multinomial logistic regression results showing odds ratios for choice of health care provider

Variables	Model 1	Model 2		

	Public	Private	Public	Private
Cost of care	0.999998*	1.000000	0.999998***	1.000000
	(0.000001)	(0.000000)	(0.000001)	(0.000001)
ln-welfare	1.097395	1.507382***	1.055778	1.382824***
	(0.144391)	(0.189305)	(0.123618)	(0.156488)
	1.007654	1.004474	0.967251*	0.957667**
	(0.005136)	(0.004955)	(0.018907)	(0.018262)
Age-squared			1.000522**	1.000549**
			(0.000257)	(0.000252)
**Insurance**				
Yes	0.239787**	0.344014*	0.259062**	0.375558*
	(0.143080)	(0.190253)	(0.147751)	(0.201183)
**Distance**				
3 to <5kms	4.056394***	1.217767	4.053354***	1.221466
	(0.902387)	(0.268491)	(0.898620)	(0.268177)
5 to <8kms	3.390711***	0.734048	3.453695***	0.766477
	(0.976709)	(0.209886)	(0.969121)	(0.214350)
8kms or more	2.364177***	0.694289*	2.415430***	0.718259
	(0.551740)	(0.151931)	(0.554126)	(0.161418)
**Education**				
Primary	1.007619	1.021970	1.281013	1.318579
	(0.199645)	(0.194639)	(0.296165)	(0.293784)
Secondary	1.451516	1.563560*	1.976700**	2.135956**
	(0.394634)	(0.411932)	(0.608186)	(0.637164)
Post-secondary	2.138766	4.097245**	2.886381	5.541655***
	(1.420548)	(2.649014)	(1.908991)	(3.585258)
**Sex**				
Female	0.941574	0.788221*	0.952178	0.777416*
	(0.128505)	(0.104117)	(0.127924)	(0.101099)
Illness days	1.006328	0.971678***	1.006223	0.971883***
	(0.011190)	(0.010252)	(0.010832)	(0.010226)
**Marital status**				
Married	1.181238	1.075821	1.484258**	1.598147***
	(0.228238)	(0.200666)	(0.274595)	(0.287170)
**Residence**				
Urban	2.135742***	1.635769***	2.140071***	1.625760***
	(0.383240)	(0.285219)	(0.367277)	(0.271062)
**Type of illness**				
Chronic	0.546275***	0.615594***	0.559583***	0.632674**
	(0.100216)	(0.109493)	(0.103431)	(0.113407)
Injury	0.265759***	0.356652***	0.275596***	0.370649***
	(0.087191)	(0.111877)	(0.090416)	(0.114988)
Other illness	0.488808***	0.526907***	0.494339***	0.535021***
	(0.076611)	(0.079554)	(0.077784)	(0.081185)
**Type of employment**				
Salaried	0.778972	0.651050**	0.796565	0.701638*
	(0.161964)	(0.132138)	(0.157865)	(0.135280)
Self-employed	0.619877***	0.646291***	0.644293***	0.703918**
	(0.099608)	(0.100997)	(0.100491)	(0.106552)
**Region**				
Eastern	2.295859***	1.314163	2.345760***	1.381031*
	(0.446893)	(0.246486)	(0.444313)	(0.252011)
Northern	1.830533***	1.151545	1.853646***	1.179882
	(0.329658)	(0.199596)	(0.333602)	(0.204272)
Western	1.888006***	1.417231*	1.952207***	1.491945**
	(0.365511)	(0.263439)	(0.392161)	(0.288307)
Household size	1.031350	1.071753**		
	(0.030577)	(0.030572)		
Sex of household head				
Male	1.089500	1.338726*		
	(0.181134)	(0.215380)		
Age of household head	0.998599	0.992167		
	(0.005776)	(0.005574)		
Constant	1.469012	0.286700	3.189228	1.195178
	(2.282153)	(0.423817)	(4.230821)	(1.531583)
Observations	9,779		9,779	
LR chi2(46)	1029		1093	
P-value	0.000		0.000	
Pseudo R-squared	0.083		0.080	

*Base category = Self-care or treatment*

Standard errors are in parentheses

*** p<0.01

** p<0.05

* p<0.1

Education positively and significantly influenced utilisation of both public and private health facilities. Holding other factors constant, having secondary or higher education increased the likelihood of using both public and private health care providers compared to having no formal education (OR= 2.00; 95%CI=1.07-3.64 and 2.89; 95%CI=0.76-10.93 respectively for government facility; and OR= 2.14; 95%CI=1.18-3.86 and 5.54; 95%CI=1.52-20.22 respectively for private facilities). A similar trend was observed with marital status where being married increased the likelihood of utilising any of the two healthcare providers relative to self-treatment.

Age had a non-linear effect and the odds of utilising public and private healthcare providers relative to self-treatment reduced significantly with increasing age. A similar trend was observed with type of employment where self-employed individuals were less likely to use both public and private healthcare providers.

Also, urban residents were 2.14 and 1.63 more likely to utilise public and private healthcare providers respectively relative to self-treatment than their counterparts in rural areas. Region of residence also significantly influenced choice of public and private health care providers. The odds of using public health facilities increased for all other regions compared to central region. Northern Uganda exhibited the lowest odds of utilising both public (OR=1.85; 95%CI=1.30-2.64) and private healthcare providers (OR=1.18; 95%CI=0.84-1.66).

Furthermore, the odds of utilising a private healthcare provider reduced significantly with increase in illness days (OR=0.97; 95%CI=0.95-0.99). Relative to self-treatment, patients with chronic/severe illness, injury or other illnesses were also less likely to use of the two health care providers than patients with fever or minor illness. Holding other factors constant, longer distances were significantly associated with more use of government health facilities relative to self-treatment.

## Discussion

The results showed that predisposing factors (age, education level, and marital status); enabling factors (household income, residence, and region) and need factors (type of illness) were significantly associated with utilisation of public and private healthcare providers. The cost of treatment was negatively associated with choice of public healthcare providers. Increasing the cost of healthcare reduces the likelihood of using public health facilities. This is consistent with findings of other studies in Tanzania 12, Kenya 13 and China 14. In Uganda, abolition of cost sharing by government increased access to health services 1.

Level of education positively influenced utilisation of both public and private healthcare providers. Individuals with secondary education and above were more likely to choose any of the two healthcare providers than those with no formal education. This may be attributed to the fact that educated individuals earn more and are therefore likely to afford to pay or better understand the benefits of utilising healthcare 8,9. This result was consistent with the findings of other studies in Ethiopia 9, Uganda 8 and Ghana 15.

Household welfare was positively associated with choice of private healthcare providers. This might be because patients from well-off households have the capacity to pay for expensive healthcare services provided in private facilities. Self-employed individuals were less likely to choose government and private healthcare providers which may be attributed to presence of a large informal sector where people earn low incomes. These findings were consistent with findings of previous studies in Ghana 7,15, Uganda 8, Tanzania 12, and Rwanda 16.

Ownership of health insurance negatively influenced choice of public health facilities. This might be due to existence of free health care in government health facilities and a low health insurance coverage. Only 5% of the individuals aged 15 years and above were covered under health insurance 5. This result was consistent with findings of a study in Jordan 17 but inconsistent with studies in Ghana 7,15, Rwanda 18, Jordan 17, and Ireland 19 who found that health insurance positively influenced utilisation of healthcare services.

Patients were more likely to visit public health care providers located far from them. Results of a study in rural China indicated that some patients may prefer to visit a more distant provider if that provider has a better reputation or patient's health status is such that only that provider can treat their illness 14. Hence, people who have special concerns and their health status is poor, distance tends to matter less and can travel longer distances.

Older patients had preference for both government and private health care providers relative to self-treatment. This finding was in agreement with prior findings in Ethiopia 9, Uganda 8, and Ghana 7,15. Further, married patients were more likely to visit public and private health care providers than their singe counterparts. This result was consistent with findings of studies in Kenya 13,20 and Jordan 17.

Controlling for all other factors, residents in urban areas were more likely to utilise public and private health facilities compared to their rural counterparts. Similarly, residents in the eastern, northern, and western regions were more likely to choose government and private health care providers compared to residents in central region. These findings were supported by studies in Uganda 8, Kenya 13, and Jordan 17 who found that geographical location significantly influenced healthcare utilisation.

For the need factors, type of illness and illness days were significant factors. Patients with long illness days were less likely to utilise public and private healthcare providers relative to self-treatment. This was a similar pattern regarding type of illness for patients with chronic illness and injury. This may be attributed to use of complementary medicine and that chronic patients obtain their drugs from shops and drug shops. These results were consistent with finding of studies conducted in rural China 14 and Uganda 8.

## Conclusions and policy implications

This study examined factors that influence choice of healthcare providers in Uganda. Findings show that higher cost of healthcare and ownership of health insurance negatively influenced choice of public health facilities. Age, level of education, marital status, urban residence, and region significantly influenced use of both public and private healthcare providers. Household welfare and paid employment were positively associated with choice of private healthcare providers. Uganda lacks a national health insurance scheme to encourage the poor and vulnerable population visit health facilities and cushion them against catastrophic expenditures. Therefore, measures need to be taken by government to decrease out of pocket payments for healthcare and enable the poor to utilise modern health facilities.

Moreover, illness days or type of illness were negatively associated with the use of private health facilities. This has noteworthy implications for the healthcare system through increased burden to providers in public health facilities and patients' ability to pay for services received. Health care providers need to modify their service models and enhance their capacity in order to adapt to different patients' needs. Given broad use of private healthcare providers, there is need for enhanced coordination between government and private sector if health policies and programs are to be successful.
